# Awareness of Hypothyroidism Complications and Compliance Among Patients With Primary Hypothyroidism in Buraidah, Saudi Arabia

**DOI:** 10.7759/cureus.114157

**Published:** 2026-08-08

**Authors:** Abdulrahman Alharbi, Elzaki M Elzaki, Omar K Alsaawi, Muath M Aldukhayel, Moayad M Alrakathi, Abdullah K Alsaawi

**Affiliations:** 1 Diabetes and Endocrinology, King Fahad Specialist Hospital, Buraidah, SAU; 2 Internal Medicine, King Fahad Specialist Hospital, Buraidah, SAU; 3 Family Medicine, Qassim University Medical City, Buraidah, SAU; 4 Family and Community Medicine, Qassim University, Buraidah, SAU; 5 Family Medicine, Second Health Cluster, Ministry of Health (MOH), Riyadh, SAU

**Keywords:** hypothyroidism, levothyroxine adherence, medication compliance, patient awareness, saudi arabia, treatment adherence barriers

## Abstract

Background

Hypothyroidism is a prevalent endocrine disorder requiring lifelong therapy, yet patient awareness of complications and adherence to levothyroxine remain variable. This study assessed awareness, medication compliance, and their socio‑demographic determinants among patients with primary hypothyroidism attending outpatient clinics at King Fahad Specialist Hospital from January 2025 to December 2025.

Methods

A cross‑sectional study was conducted among 209 adult patients diagnosed with primary hypothyroidism. Awareness of hypothyroidism complications, compliance, and reasons for nonadherence to medication were assessed using a structured questionnaire. Associations among awareness, compliance, and socio-demographic variables were analyzed using descriptive statistics, the Mann-Whitney Z-test, and Spearman's correlation. Multiple linear regression analysis was performed to determine significant predictors of awareness score.

Results

The patients were predominantly female (195, 93.3%) and married (169, 80.9%). Awareness of hypothyroidism complications and compliance were both moderate (mean: 6.76±2.21 and 3.04±1.25). Forgetfulness was the most commonly reported reason for non-compliance (187, 89.5%), followed by polypharmacy (80, 38.3%). A strong positive correlation was observed between awareness and compliance (rs=0.585; p<0.001). A significantly higher level of awareness was observed among females (p=0.009), those with higher education (p=0.018), and those diagnosed with hypothyroidism for more than 10 years (p=0.004). Except for marital status, which was associated with higher compliance among married individuals (p=0.025), no significant associations were found between compliance and the rest of the socio‑demographic variables. Multiple linear regression identified gender (β=1.858; 95% CI: 0.681-4.702; p=0.002), higher educational level (β=0.962; 95% CI: 0.267-1.657; p=0.007), and longer duration of hypothyroidism (β=0.925; 95% CI: 0.321-1.529; p=0.003) as independent predictors of higher awareness, after adjusting for age and marital status.

Conclusion

Patients with hypothyroidism showed moderate awareness of complications and moderate medication compliance. Higher awareness was associated with female sex, higher education, and longer disease duration, although this may be influenced by the predominance of females in the sample. No significant associations were found between compliance and most socio-demographic variables, except higher compliance among married individuals, which may reflect sample distribution rather than an independent effect. A positive correlation was observed between awareness and compliance, suggesting that higher awareness is associated with better adherence. Forgetfulness and polypharmacy were the main barriers to compliance, supporting the need for targeted educational and compliance-support strategies.

## Introduction

Hypothyroidism is a common endocrine disorder in which the thyroid gland does not make enough thyroid hormone. It causes a metabolic disturbance that affects many systems. It is estimated to affect 4-10% of the world's population and is more common in females and the elderly [1]. Recent regional analyses have demonstrated a considerable burden of thyroid dysfunction in Middle Eastern populations [2]. Recent epidemiological reviews in Saudi Arabia have estimated the prevalence of overt and subclinical hypothyroidism to be between 11% and 15%, thus highlighting the importance of this chronic condition that requires long-term management [3,4].

Successful treatment relies on strict adherence to levothyroxine therapy to maintain euthyroid status and avoid complications like dyslipidemia, weight gain, cardiovascular disease, infertility, menstrual irregularities, neurocognitive impairment, and myxedema coma [5,6]. Knowledge of these complications is important for encouraging the patient to comply with therapy and regular follow-up.

Nevertheless, studies have shown that compliance with levothyroxine therapy can be inconsistent, often influenced by patients' knowledge of the disease, beliefs about long‑term treatment, dosing requirements, and understanding of the consequences of missed doses [7]. Knowledge of the potential complications of hypothyroidism is an important factor in the development of compliance behaviors, as patients who are aware of the risk posed by uncontrolled disease may be more likely to follow treatment recommendations [8]. Studies of medication adherence among patients with chronic diseases in Saudi Arabia have shown diverse patterns influenced by health literacy, cultural beliefs, and patient-provider communication [9].

Emerging literature also highlights that untreated or inadequately managed hypothyroidism can affect multiple physiologic systems, reinforcing the importance of sustained and appropriate long‑term therapy [10]. Compliance with medication remains an important aspect of the management of hypothyroidism. Levothyroxine should be taken daily on an empty stomach and apart from substances that interfere with its absorption to ensure optimal absorption [5]. Non-compliance has been linked to persistent symptoms, increased thyroid-stimulating hormone (TSH) levels, higher healthcare utilization, and lower quality of life [7]. International studies have reported compliance rates of 40-80%, depending on health literacy, socioeconomic status, and support from the healthcare system [8].

Research in Middle Eastern and Saudi populations has shown that many patients have inadequate knowledge of hypothyroidism and its complications, leading to irregular medication use [2,7]. A study in the Qassim region highlighted the role of illness perception in compliance behaviors among hypothyroid patients [7]. However, region-specific evidence on compliance and awareness of hypothyroidism complications remains limited. Identifying gaps in awareness and compliance will support targeted educational interventions and improve chronic disease management in Saudi Arabia. Hence, this study aimed to evaluate compliance among patients with primary hypothyroidism, assess their awareness of disease-related complications, determine the correlation between awareness and compliance, and identify the socio-demographic determinants influencing these outcomes in the Qassim region.

## Materials and methods

Study design, setting, and sampling

This study employed a cross-sectional design using convenience sampling to evaluate awareness of hypothyroidism complications and compliance among adult patients with hypothyroidism attending outpatient clinics at King Fahad Specialist Hospital in Buraidah, Saudi Arabia. Data were collected from January 2025 to December 2025 using a structured questionnaire conducted via telephone interviews after obtaining participants' consent. A total of 223 eligible patients were contacted, of whom 209 agreed to participate (response rate: 93.7%).

Inclusion and exclusion criteria

Patients included in the study had primary hypothyroidism diagnosed according to the diagnostic criteria of the American Thyroid Association and the American Association of Clinical Endocrinologists. Primary hypothyroidism was defined as elevated TSH levels above the laboratory reference range (>4.0 mIU/L) and low free T4 levels (<9 pmol/L) and often with clinical signs or symptoms of hypothyroidism [11]. Patients were excluded if they had subclinical hypothyroidism without the initiation of levothyroxine therapy, had cognitive impairment (e.g., dementia or other mental disabilities) that could compromise the reliability of questionnaire responses, were younger than 18 years of age, or had incomplete hospital records. Patients excluded from the analyses were not included in the statistical evaluation. All statistical analyses were conducted using the final analytical sample, and no imputation procedures were performed.

Sample size calculation

The sample size was calculated using a single‑proportion formula based on the expected prevalence of adequate adherence to levothyroxine among patients with hypothyroidism. Assuming an anticipated adherence proportion of approximately 50% (to maximize sample size), a 95% confidence level, and a margin of error of 7%, the minimum required sample size was estimated at around 190 participants. The calculated sample was increased by an extra 10% to account for possible non-response, incomplete data, and exclusions, giving a final target sample size of 209 patients. A fixed sample size of 209 was considered adequate to provide sufficient statistical power to detect meaningful associations between awareness of complications and adherence.

Data collection instrument 

Data were collected using a structured questionnaire (see Appendices). The questionnaire was interviewer-administered through telephone interviews conducted by the study investigators. All questions were read to participants as written in the questionnaire, and responses were recorded directly by the interviewer using a standardized approach. The instrument contained demographic variables such as age, gender, education, occupation, and duration since diagnosis. It also included a 14-item awareness scale covering complications of uncontrolled hypothyroidism, a five-item medication compliance scale reflecting recommended levothyroxine practices, and a checklist of common reasons for non-compliance. Awareness items addressed a broad range of hypothyroidism‑related complications, including mood disturbances, cognitive dysfunction, peripheral neuropathy, severe confusion, pericardial effusion, dyslipidemia, cardiovascular disease, weight gain, anemia, menstrual irregularities, infertility, pregnancy‑related risks, and congenital hypothyroidism. Adherence items evaluated outpatient follow‑up attendance, regularity of thyroid function testing, daily levothyroxine intake, fasting administration practices, and avoidance of food immediately after medication. Additional items explored reasons for nonadherence to medication, including perceived improvement or lack of improvement, pill burden, laboratory normalization, fear of side effects, insurance limitations, forgetfulness, and difficulty complying with fasting requirements.

The questionnaire was developed by adapting items from previously published studies that assessed patient awareness of hypothyroidism‑related complications and adherence to levothyroxine therapy, ensuring alignment with established constructs in the Saudi population and international literature [12-14]. Content validity was established through expert review by specialists, who evaluated item relevance, clarity, and cultural appropriateness. The instrument was then pilot‑tested on 20 adult hypothyroid patients. Feedback from the pilot phase led to minor refinements in wording and response options, particularly in the awareness and compliance domains, to improve clarity and reduce ambiguity. Data from the pilot test were not included in the final analysis.

Each item was scored as either correct/appropriate (1 point) or incorrect/inappropriate (0 points), yielding maximum possible scores of 14 points for awareness and 5 points for compliance. Higher scores indicated greater awareness and better medication adherence. To categorize participant performance, total scores for each domain were subdivided into three levels using the 50% and 75% cutoff points recommended by Qutob et al. [15]. Scores below 50% of the total possible score were classified as poor, scores between 50% and 75% were classified as moderate, and scores above 75% were classified as good. This scoring framework allowed for the standardized interpretation of awareness and compliance levels within the study population.

Statistical analysis

Descriptive statistics were generated using IBM SPSS Statistics for Windows, Version 26.0 (IBM Corp., Armonk, New York, United States). Categorical variables were summarized as frequencies and percentages, whereas continuous variables were presented as mean±standard deviation. The normality of awareness and compliance scores was assessed using the Kolmogorov-Smirnov test, which confirmed a non‑normal distribution for all composite scores. Therefore, a non‑parametric test was applied. Comparisons between two independent groups were performed using the Mann-Whitney Z-test. The correlation between awareness and compliance was examined using Spearman's rank correlation coefficient. A p‑value of <0.05 was considered statistically significant. To identify independent predictors of awareness of hypothyroidism, a multiple linear regression analysis was performed. Variables that showed significant differences in awareness scores in the comparison analyses were entered into the regression model, adjusting for age and marital status.

## Results

The socio-demographic characteristics of the 209 patients with hypothyroidism are presented in Table 1. The age distribution showed that the largest proportion of patients, 92 (44%), were aged >40 years, followed by 67 (32.1%) aged 31-40 years. The sample consisted mainly of females (195, 93.3%) who were married (169, 80.9%). Educational attainment was generally high, with most participants holding a bachelor's degree (126, 60.3%). Regarding disease duration, 66 (31.6%) had been diagnosed with hypothyroidism for 6-10 years, while 27 (12.9%) had been living with the condition for more than 15 years.

**Table 1:** Socio-demographic characteristics of the patients with hypothyroidism (n=209) Results are expressed as numbers and percentages, N (%).

Study variables	N (%)
Age group	
18-30 years	50 (23.9%)
31-40 years	67 (32.1%)
>40 years	92 (44%)
Gender	
Male	14 (6.7%)
Female	195 (93.3%)
Marital status	
Single	40 (19.1%)
Married	169 (80.9%)
Educational level	
Primary	2 (1%)
Intermediate	20 (9.6%)
Secondary	57 (27.3%)
Bachelor's degree	126 (60.3%)
Postgraduate	4 (1.9%)
Duration of hypothyroidism since diagnosis	
1-5 years	57 (27.3%)
6-10 years	66 (31.6%)
11-15 years	59 (28.2%)
>15 years	27 (12.9%)

Awareness of hypothyroidism complications was highest for weight gain (173, 82.8%) and menstrual irregularities (162, 77.5%) and lowest for pericardial effusion (47, 22.5%). The total mean awareness score was 6.76±2.21. Nearly half of the patients were considered to have moderate awareness (104, 49.8%), followed by poor (63, 30.1%) and good (42, 20.1%). Compliance patterns indicated higher adherence for taking levothyroxine on an empty stomach (139, 66.5%) and 30-60 minutes before food (138, 66%), whereas lower adherence was noted for routine thyroid function testing every 6-12 months (119, 56.9%). The total mean compliance score was 3.04±1.25, with compliance levels distributed across poor (79, 37.8%), moderate (58, 27.8%), and good (72, 34.4%) (Table 2).

**Table 2:** Assessment of awareness of hypothyroidism complications and compliance among patients with hypothyroidism (n=209) Results are expressed as numbers and percentages, N (%), or mean±standard deviation.

Awareness of complications and compliance items	Value
Awareness of complications items	
Weight gain (Yes)	173 (82.8%)
Menstrual irregularities (Yes)	162 (77.5%)
High lipid profile (Yes)	140 (67%)
Severe confusion (Yes)	132 (63.2%)
Anemia (Yes)	118 (56.5%)
Depression and mood disturbances (Yes)	103 (49.3%)
Increased risk of miscarriage, preeclampsia, and preterm birth in pregnancy (Yes)	95 (45.5%)
Infertility (Yes)	89 (42.6%)
Atherosclerosis and coronary artery disease (Yes)	78 (37.3%)
Cognitive dysfunction ("brain fog", memory issues) (Yes)	75 (35.9%)
Heart failure (Yes)	70 (33.5%)
Congenital hypothyroidism in the fetus (Yes)	67 (32.1%)
Peripheral neuropathy (Yes)	66 (31.6%)
Pericardial effusion (Yes)	47 (22.5%)
Total awareness score (mean±SD)	6.76±2.21
Level of awareness	
Poor	63 (30.1%)
Moderate	104 (49.8%)
Good	42 (20.1%)
Compliance items	
Do you take the medication on an empty stomach? (Yes)	139 (66.5%)
Do you take the medication 30-60 minutes before having any food or other medications? (Yes)	138 (66%)
How often do you visit your physician? (Every 6-12 months)	120 (57.4%)
Do you frequently miss taking your medication >2 times per week? (No)	120 (57.4%)
How often do you do a thyroid function test? (Every 6-12 months)	119 (56.9%)
Total compliance score (mean±SD)	3.04±1.25
Level of compliance	
Poor	79 (37.8%)
Moderate	58 (27.8%)
Good	72 (34.4%)

**Figure 1:**
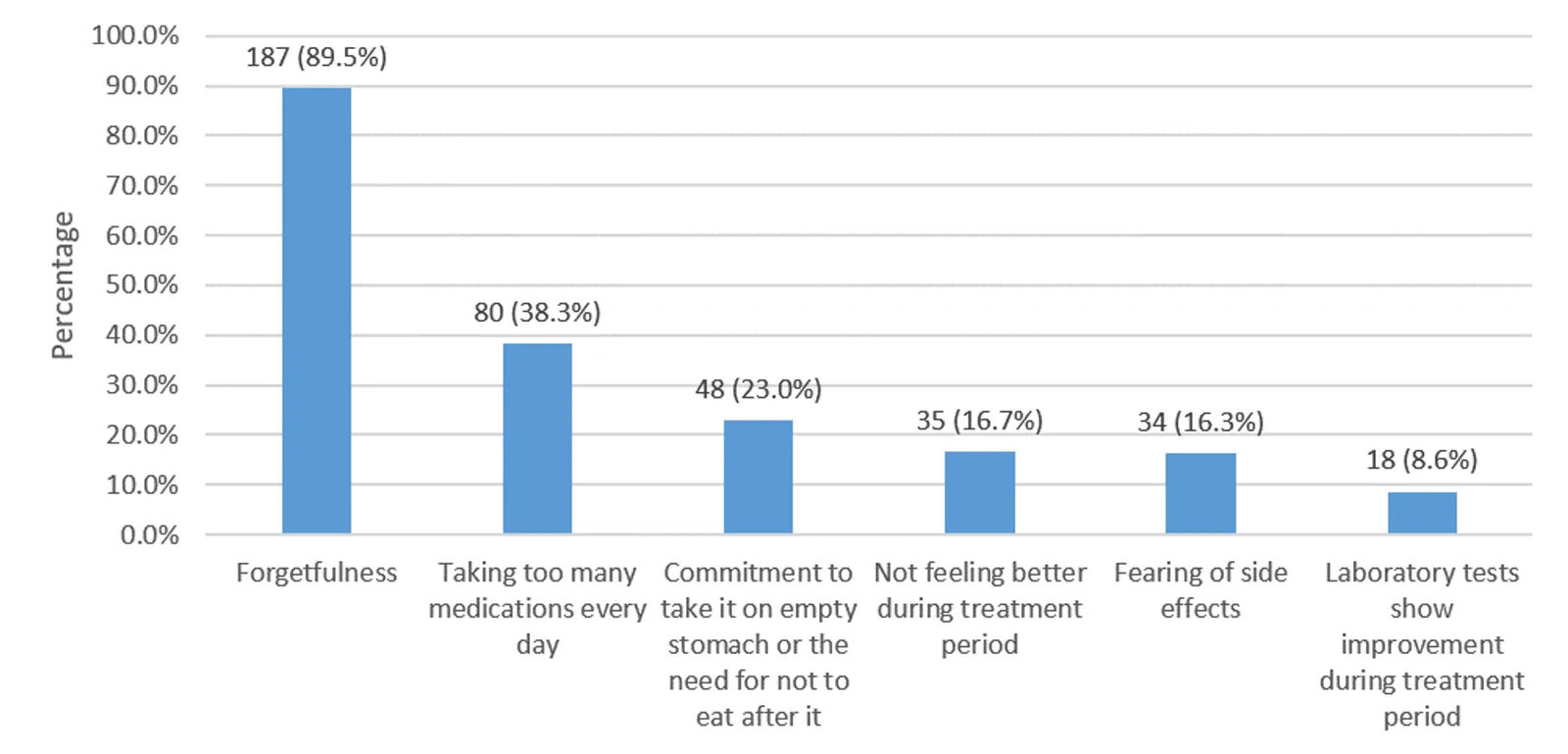
Reasons for non-compliance with medications (n=209)

Figure 1 shows that the most frequently reported reason for non‑compliance with medication was forgetfulness (187, 89.5%), followed by the perception of taking too many medications every day (80, 38.3%) and commitment to taking levothyroxine on an empty stomach or avoiding food afterward (48, 23%).

**Figure 2:**
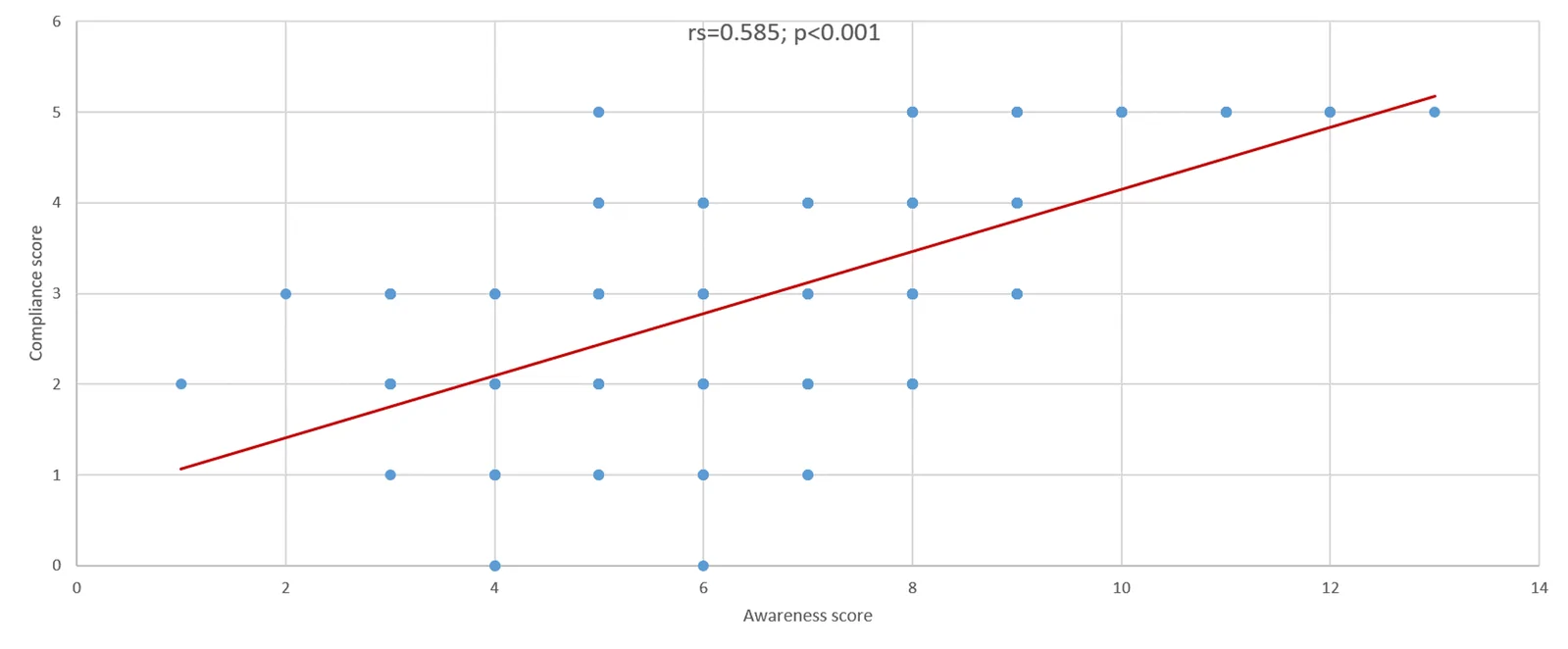
Spearman's correlation between awareness and compliance scores (n=209)

The scatter plot in Figure 2 shows a strong, statistically significant positive correlation between awareness and compliance scores, as indicated by Spearman's rank correlation coefficient (rs=0.585; p<0.001). The data points are trending upward, indicating that higher awareness scores are associated with higher compliance scores, suggesting a significant association between patients' knowledge of hypothyroidism complications and compliance. The pattern suggests that higher awareness scores are associated with higher compliance scores.

Table 3 presents several significant associations between awareness scores and socio‐demographic characteristics. Females had significantly higher awareness (6.89±2.14) than males (5.00±2.48), with a significant difference (Z=2.615; p=0.009). A significant association was also found between awareness and educational level, with those who had a bachelor's degree or higher (7.08±2.21) having higher awareness than those with secondary education or below (6.24±2.13) (Z=2.367; p=0.018). Duration of hypothyroidism was significantly associated with awareness (Z=2.844; p=0.004): those diagnosed for >10 years had higher awareness (7.34±2.27) than those diagnosed for ≤10 years (6.36±2.08). No significant differences were found in age group (p=0.741) or marital status (p=0.069).

**Table 3:** Association between awareness and the socio-demographic characteristics among patients with hypothyroidism (n=209) Results are expressed as mean ± standard deviation. §P-value has been calculated using the Mann-Whitney Z-test. **Significant at p<0.05 level.

Factor	Awareness score (14) mean±SD	Z-test	P-value^§^
Age group			
<40 years	6.76±2.11	0.330	0.741
≥40 years	6.76±2.34		
Gender			
Male	5.00±2.48	2.615	0.009**
Female	6.89±2.14		
Marital status			
Single	6.18±2.37	1.821	0.069
Married	6.89±2.15		
Educational level			
Secondary or below	6.24±2.13	2.367	0.018**
Bachelor's degree or higher	7.08±2.21		
Duration of hypothyroidism since diagnosis			
≤10 years	6.36±2.08	2.844	0.004**
>10 years	7.34±2.27		

Table 4 shows that most socio-demographic characteristics were not statistically significantly related to compliance scores, including age (p=0.697), gender (p=0.925), educational level (p=0.525), and duration of hypothyroidism (p=0.672). A significant association was observed with marital status, with married individuals having higher compliance (3.13±1.23) than single individuals (2.68±1.29) (Z=2.238; p=0.025).

**Table 4:** Association between compliance and the socio-demographic characteristics of the patients with hypothyroidism (n=209) Results are expressed as mean±standard deviation. §P-value has been calculated using the Mann-Whitney Z-test. **Significant at p<0.05 level.

Factor	Compliance score (5) mean±SD	Z-test	P-value^§^
Age group			
<40 years	3.03±1.18	0.389	0.697
≥40 years	3.06±1.33		
Gender			
Male	3.07±0.99	0.094	0.925
Female	3.04±1.27		
Marital status			
Single	2.68±1.29	2.238	0.025**
Married	3.13±1.23		
Educational level			
Secondary or below	2.96±1.29	0.636	0.525
Bachelor's degree or higher	3.09±1.23		
Duration of hypothyroidism since diagnosis			
≤10 years	2.98±1.26	0.424	0.672
>10 years	3.13±1.23		

Awareness of hypothyroidism was significantly influenced by gender, educational attainment, and duration since diagnosis after adjusting for age and marital status (Table 5). Female participants demonstrated higher awareness scores, with gender showing the strongest effect (β=1.858; 95% CI: 0.681-4.702; p=0.002). Higher educational level was also an independent predictor (β=0.962; 95% CI: 0.267-1.657; p=0.007). Additionally, longer duration of hypothyroidism since diagnosis was associated with greater awareness (β=0.925; 95% CI: 0.321-1.529; p=0.003). 

**Table 5:** Multiple linear regression analysis to determine the significant predictors of awareness of hypothyroidism (n=209) Adjusted for age and marital status. **Significant at p<0.05 level.

Factor	Unstandard β	Std. error	Standard coefficient β	95% CI	P-value
Gender	1.858	0.597	0.211	0.681-4.702	0.002**
Educational level	0.962	0.352	0.211	0.267-1.657	0.007**
Duration of hypothyroidism since diagnosis	0.925	0.306	0.206	0.321-1.529	0.003**

## Discussion

The socio‑demographic profile of the study population reflects patterns commonly reported in regional and international hypothyroidism research. Nearly half of the patients were older than 40 years, consistent with the well‑established increase in hypothyroidism prevalence with advancing age [1]. The overwhelming predominance of females (93.3%) aligns with established epidemiologic evidence showing that thyroid disorders disproportionately affect women, with global female‑to‑male ratios of approximately 5:1 to 10:1 as reported by Vanderpump [1], while regional studies similarly confirm a higher burden of thyroid disease among women in Middle Eastern populations [2-4]. Comparable female predominance has also been documented in Jordan, Oman, Lebanon, and India, reinforcing the universal gender disparity in thyroid disease burden [16-19]. The high proportion of married and well‑educated participants is similar to findings from Saudi‑based studies [7,12].

General awareness of hypothyroidism complications was moderate (mean score: 6.76±2.21). The most recognized complications of hypothyroidism were weight gain and menstrual irregularities. Our results are in line with previous Saudi and regional studies that have shown high awareness of common, visible, or personally experienced symptoms, especially among women [7,14,15]. Similar trends have been observed in North India and the western region of Saudi Arabia, where patients had higher awareness about overt symptoms but poor recognition of systemic or cardiovascular complications [19,20]. On the contrary, knowledge of less-known or clinically complex complications, such as pericardial effusion, was low. This finding is consistent with previous evidence showing that patients are rarely aware of cardiovascular or metabolic consequences of hypothyroidism [6,10]. Low awareness of long-term complications is also reported in studies from Oman and Jordan, indicating this knowledge gap exists in different populations [16,17]. Compared to the Riyadh-based study by Qutob et al. [15], who found generally low awareness of thyroid complications, our cohort showed better awareness of several complications. This difference might be due to sample characteristics: the percentage of long-term diagnosed patients was higher in our study than in other studies, and these patients might have had more clinical encounters and education over time.

The distribution of levels of awareness is similar to the findings in Delhi and Al Qassim, where moderate knowledge was also predominant [7,8], with almost half of the cohort being moderate. Our percentage of patients with good awareness (42, 20.1%) is, however, higher than that reported in previous Saudi community‑based studies [14,15]. This may be due to better public health messaging, increased access to digital health information, or greater exposure to healthcare services among patients with established disease. Studies from Jordan and India have recently reported similar improvements in awareness, with digital health literacy and increased screening initiatives helping improve patient knowledge [16,19,21].

Patterns of compliance showed moderate overall compliance (mean score: 3.04±1.25), with the highest adherence for taking levothyroxine on an empty stomach and before food. This is consistent with compliance patterns reported in Madinah and India, where patients were more likely to follow simple, routine-based instructions than to engage in long-term monitoring behaviors [8,12]. Similarly, research in Oman, Lebanon, and Jordan has reported increased adherence to daily dosing instructions but decreased adherence to follow-up testing and dose adjustments, which may point to a global trend of partial adherence to hypothyroidism management [16-18]. The relatively lower rate of adherence to routine thyroid function testing (119, 56.9%) is consistent with findings from national chronic disease adherence studies, which show that laboratory follow-up is often given lower priority due to cost, inconvenience, or perceived stability of symptoms [9]. Our cohort had better compliance than the study from Madinah, which reported lower overall adherence [12]. This difference may be explained by higher educational attainment in our sample and a larger proportion of patients with long-standing disease, who may have developed more stable medication routines. Similar associations between education, disease duration, and adherence have been reported in Oman, India, and South Asia [17,19,22].

Forgetfulness emerged as the most common reason for non‑compliance (187, 89.5%), consistent with findings from Saudi Arabia and India, where forgetfulness is repeatedly identified as the leading barrier to adherence in chronic endocrine conditions [7-9,12]. This barrier is also widely reported in Jordan, Oman, and Lebanon, where forgetfulness remains the primary cause of missed doses despite adequate knowledge of the importance of treatment [16-18]. Other barriers, such as pill burden and difficulty with fasting recommendations, are consistent with previous literature reporting that the timing and drug interaction profile of levothyroxine can have a negative impact on adherence, especially in patients with multiple comorbidities and complex medication regimens [5,10]. Similarly, studies in Saudi Arabia, India, and Indonesia found fasting‑related dosing, food interactions, and polypharmacy to be major contributors to poor adherence [20,22,23].

The main finding of this study is a strong positive correlation between awareness and compliance (rs=0.585; p<0.001), indicating that patients with higher awareness scores also demonstrated higher compliance scores with levothyroxine therapy. This is consistent with previous studies that have demonstrated that perceptions of illness, knowledge about the consequences of the disease, and knowledge about the benefits of treatment have a significant impact on compliance behaviors [7,8,12]. Similar associations have been reported in Jordan, Oman, and India, where more awareness and understanding of hypothyroidism were strongly associated with better compliance and medication-taking behavior [16,17,19,21]. The magnitude of correlation in our study is stronger than that reported in earlier Saudi cohorts, which may reflect the higher baseline awareness in our sample or differences in measurement tools.

Awareness was significantly associated with gender, education, and duration of hypothyroidism. Females demonstrated higher awareness than males, consistent with epidemiologic patterns showing that women are more engaged in thyroid‑related health information and more likely to experience symptomatic disease [1,4,7]. However, this gender difference should be interpreted with caution because the sample was predominantly female, leaving relatively few males and limiting statistical power to detect true differences between groups. Increased awareness was also associated with higher educational attainment, consistent with the finding that health literacy plays an important role in understanding endocrine disorders [7,8,15]. Higher levels of education are consistent predictors of better thyroid-related knowledge in India, Lebanon, and Jordan, as shown in similar studies [16,18,19,21]. Patients with a diagnosis for more than 10 years had higher awareness, likely because of cumulative exposure to clinical counseling, repeated follow-up visits, and personal experience with disease progression. These results are in agreement with previous studies indicating that patients with longer disease duration have better knowledge and self-management skills [7,12,17].

On the contrary, compliance was not significantly associated with most socio‑demographic characteristics, with the exception of marital status. Compliance scores were higher among married individuals, a finding also reported in studies of adherence to chronic disease treatment in Saudi Arabia [9]. However, this finding should be interpreted with caution, as the sample was predominantly married and there were relatively few single participants available for comparison, thereby limiting the statistical power of subgroup analyses. Shared household routines and spousal involvement in promoting medication adherence, as well as evidence of the importance of social support, are also found in Jordan and India [16,19]. These contextual factors, from family member reminders to structured daily routines, may help explain why married people tend to be better at following through than their single counterparts. No significant associations were found with age, gender, education, or disease duration, suggesting that adherence behaviors may be more influenced by psychosocial and behavioral factors, such as forgetfulness, perceived burden, and daily routines, than by demographic characteristics alone. Similarly, studies conducted in Oman, Lebanon, and Indonesia [17,18,22] have highlighted that behavioral and cognitive predictors are more important than demographic predictors for adherence.

Overall, the findings highlight the need for targeted patient education, particularly on more unfamiliar complications, and for regular monitoring of thyroid function. The strong correlation observed suggests that higher awareness is associated with better adherence. Interventions tailored to forgetfulness, such as reminder systems, simplified dosing routines, and counseling about the consequences of missed doses, may further improve compliance and long-term outcomes. Structured counseling, digital reminders, and pharmacist-led interventions have been shown to improve compliance and patient understanding of hypothyroidism in studies from Jordan, India, and Lebanon [16,18,19,21].

Strengths of the study

This study has several strengths. It addresses an important clinical question in a region with a relatively high burden of hypothyroidism and provides region-specific data on patient awareness and medication compliance. The study included an adequate sample size, enhancing the reliability of the analyses. In addition, the findings are presented comprehensively, with detailed analyses of awareness and compliance and their associations with socio-demographic characteristics. Finally, the findings were interpreted in the context of both regional and international literature, improving the relevance and generalizability of the study within similar healthcare settings.

Limitations of the study

There are several limitations to this study that should be considered when interpreting the findings. First, the cross-sectional design limits the ability to infer causality between awareness, compliance, and socio-demographic factors. Second, the use of convenience sampling may have introduced selection bias and limited the representativeness of the study population. Third, the sample was highly unbalanced by gender, with females representing 93.3% of the participants. Although this reflects the known epidemiology of hypothyroidism, the small number of male participants limits the statistical power to detect meaningful gender-based differences in awareness or compliance. Similarly, the predominance of married individuals (80.9%) resulted in relatively few single participants, which may have reduced the robustness of comparisons by marital status, particularly since marital status was significantly associated with compliance. Fourth, data were collected through telephone-based self-reporting, which may have introduced recall and social desirability biases, potentially leading to the overestimation of compliance and awareness levels. Fifth, the study was conducted at a single tertiary care hospital, which may limit the generalizability of the findings to primary care settings or populations with lower health literacy. Furthermore, although the questionnaire underwent expert-based content validation and pilot testing before implementation, additional psychometric evaluation, including assessments of internal consistency and construct validity, may further strengthen the instrument in future studies. In addition, several factors that may influence medication compliance were not assessed, including depression, health literacy, socioeconomic status, physician counseling frequency, and family support, leaving room for residual confounding. Finally, clinical parameters such as thyroid function tests were not incorporated, preventing correlation between self-reported compliance and biochemical control.

## Conclusions

The findings of this study reveal that patients with hypothyroidism have moderate awareness of complications of the disease and moderate adherence to levothyroxine therapy. Several socio-demographic factors significantly affect these outcomes. Higher awareness scores were substantially associated with females, patients with higher levels of education, and those with a diagnosis for more than 10 years, underscoring the importance of health literacy and long-term experience with the condition in determining patient understanding. The observed association between awareness and compliance highlights the potential role of patient education as a component of long-term disease management strategies. However, further studies are required to determine whether educational interventions directly improve adherence. Adherence is still challenged by key barriers such as forgetfulness, pill burden, and dose issues due to fasting, suggesting the need for specialized patient-centered therapies. Better educational initiatives, increased follow-up, and addressing behavioral barriers offer potential to improve clinical outcomes for those living with hypothyroidism.

## Appendices

Questionnaire

Demographics

1.    Age (a)  To be written by the participant in number-of-years format

2.    Gender (a)   Male (b)   Female

3.    Marital status (a)   Married (b)   Single

4.    Education (a)   Primary (b)   Intermediate (c)   Secondary (d)   Bachelor's degree (e)   Postgraduate degree

5.    Since when have you been diagnosed? (a)   To be written by the participant in number-of-years format

Awareness About Uncontrolled Hypothyroidism Complications

(1)   What are the complications of uncontrolled hypothyroidism that you know? (Multiple choices allowed) 1.     Depression and mood disturbances* 2.    Cognitive dysfunction ("brain fog", memory issues)* 3.    Peripheral neuropathy* 4.    Severe confusion* 5.    Pericardial effusion* 6.    High lipid profile* 7.    Atherosclerosis and coronary artery disease* 8.    Heart failure* 9.    Weight gain* 10.  Anemia* 11.   Menstrual irregularities* 12.  Infertility* 13.  Increased risk of miscarriage, preeclampsia, and preterm birth in pregnancy* 14.  Congenital hypothyroidism in the fetus*

Hypothyroidism Compliance

1. How often do you visit your physician? - Every 6-12 months* - >12 months

2. How often do you do thyroid function tests? - Every 6-12 months* - >12 months

3. Do you frequently miss taking your medication >2 times per week? - Yes - No*

4. Do you take the medication on an empty stomach? - Yes* - No

5. Do you take the medication daily 30-60 minutes before having any food or other medications? - Yes* - No

* indicates the correct answer.

Reasons for Non-compliance With Medication

1.    Not feeling better during the treatment period 2.    Taking too many medications every day 3.    Laboratory tests show improvement during the treatment period 4.    Fear of side effects 5.    Forgetfulness 6.    Commitment to take it on an empty stomach or the need not to eat after it
